# The Effect of Physical Activity Interventions Carried Out in Outdoor Natural Blue and Green Spaces on Health Outcomes: A Systematic Review

**DOI:** 10.3390/ijerph191912482

**Published:** 2022-09-30

**Authors:** Sofia Marini, Mario Mauro, Alessia Grigoletto, Stefania Toselli, Pasqualino Maietta Latessa

**Affiliations:** 1 Department for Life Quality Studies, University of Bologna, 47921 Rimini, Italy; 2 Department of Biomedical and Neuromotor Sciences, University of Bologna, 40126 Bologna, Italy

**Keywords:** blue exercise, green exercise, healthy adults, blue–green space setting, natural outdoor environment, physical activity intervention, outdoor exercise, health promotion

## Abstract

In the last few years, interest about the natural environment and its influences on health conditions has been growing. In particular, physical activity interventions carried out in blue and green environment are being investigated as a potential strategy to increase health outcomes in people with and without chronic conditions. Many recent studies reported positive results, but a high number of these studies were focused on people with mental or physical disorders. In this scenario, the present systematic review, conducted according to the PRISMA statement, was aimed at investigating the existing evidence regarding the effects of physical activity interventions carried out in green–blue space settings involving healthy people. A literature search was performed through PubMed, Cochrane, Cinahl, and Psychinfo, and the quality of each study was assessed. Out of 239 identified articles, 75 full texts were screened. Six eligible studies showed an improvement in health outcomes, such as well-being, mood, and physical performance, in the experimental group compared with the control group. No exhaustive conclusion can be drawn based on available evidence. However, this systematic review highlighted the need to extend this kind of intervention to reveal more robust evidence that green and blue exercises benefit health.

## 1. Introduction

Green spaces can be defined as open areas of ground, covered by vegetation, which includes parks and gardens [1]. On the other hand, blue spaces are accessible settings principally consisting of water such as rivers and lakes [2]. In recent years, there has been an increase in the literature about these kinds of natural environments: green and blue spaces [1]. This is linked to the worldwide growing urbanization [3]. One of the major challenges for the future will be to create cities on a human scale that can be habitable ensuring wholesome conditions, and to achieve this will be fundamental to safeguarding natural environments. Recent systematic reviews suggest that people, especially those with mental or physical disorder, can obtain health benefits if they use and are exposed to natural outdoor environments [4,5]. Therefore, access to green and blue spaces, such as beaches and gardens, provides opportunities to support and promote public health and well-being [6,7].

There are an increasing number of studies with the aim of estimating the impact of access and exposure to neighborhood green and blue spaces on the risk of mental health conditions and the opportunity for promoting well-being [8,9]. Up-to-date research indicates that the benefits may be different due to the population groups, context, and health outcome [10,11]. Moreover, the mechanisms that justify the connection between the natural environment and well-being are still unclear [12,13]. Even if some studies proposed different mechanisms to explain this relationship, one of these mechanisms is the opportunity to perform physical activity (PA) [14,15,16]. Adequate PA levels are essential, given that PA is a fundamental aspect of human health [17]. Indeed, conducting an active lifestyle contributes to the prevention of noncommunicable diseases such as stroke, diabetes, hypertension, overweight, and obesity. Moreover, it improves the well-being, mental health, and quality of life [17]. Despite this evidence, a great part of the population is still inactive. The use of green and blue spaces can help people to achieve the right amount of PA. Indeed, it can facilitate PA, social interaction, and contact with nature, providing multiple health benefits [8,18,19,20,21,22]. Experimental studies suggested that outdoor exercise can be a possible alternative to indoor exercise and that exposure to a natural environment is connected to a higher amount of PA and a lower mortality rate [23,24,25]. In addition, it seems that performing PA in a natural environment can have supplementary benefits in comparison with PA performed in an indoor environment [26].

Therefore, evidence of such health benefits might be of high relevance for healthcare professionals, urban planners, and policymakers, who can help translate available evidence into interventions and policies targeted to improve health. However, the knowledge base is limited to the green and blue spaces evaluation of exposures or nearness separately considering green or blue or involving a nonhealthy population. In such a scenario, the aim of the present systematic review was to investigate the existing evidence regarding the effects of PA interventions performed in a natural environment involving healthy people (aged ≥ 18 years).

## 2. Materials and Methods

### 2.1. Data Sources and Search Strategy

The present systematic review was prepared in accordance with PRISMA recommendations and guidelines [27]. The protocol was registered in the International Prospective Register of Systematic Reviews (PROSPERO).

Table 1 summarizes the Patients, Interventions, Comparators, Outcomes, Time, and Setting (PICOTS) criteria drafted to address the primary search aim.

A systematic literature search of the main coherent databases according to the aim of this paper, MEDLINE (PubMed), Cochrane Central Register of Controlled Trials (Central), CINAHL (EBSCO), and PSYCHINFO (EBSCO), from April 2022 up to May 2022 was conducted to identify all published articles about PA interventions carried out in green and blue spaces and relative effects in terms of physical fitness, quality of life, physical performance, and anthropometric characteristics focusing on healthy adults.

Only randomized controlled trials (RCTs), case reports, clinical trials, observational studies, and clinical trials for which the full text was available were included. In addition, only human subjects were included, and we decided to put a 10-year publication date limit. Search strategies (strings adapted when necessary in order to fit the specific search requirements of each database) used the following Boolean expression, keywords, and terms (terms mainly chosen from papers related to the topic and mesh database): (“Exercis*” OR “Physical Activity” OR “Activities, Physical” OR “Activity, Physical” OR “Physical Activities” OR “Exercise, Physical” OR “Exercises, Physical” OR “Physical Exercise” OR “Physical Exercises” OR “Acute Exercise” OR “Acute Exercises” OR “Exercise, Acute” OR “Exercises, Acute” OR “Exercise, Isometric” OR “Exercises, Isometric” OR “Isometric Exercises” OR “Isometric Exercise” OR “Exercise, Aerobic” OR “Aerobic Exercise” OR “Aerobic Exercises” OR “Exercises, Aerobic” OR “Exercise Training” OR “Exercise Trainings” OR “Training, Exercise” OR “Trainings, Exercise”) AND (“Outdoor Exercis*” OR “Outdoor Fitness” OR “Outdoor Physical Activity” OR “Natural Environment Exercise” OR “Blue Space Physical Activity” OR “Green Urban Space Exercis*” OR “Green Urban Space Physical Activity” OR “Outdoor Training” OR “Outdoor Circuit Training” OR “Outdoor Resistance Training” OR “Outdoor High Intensity Training” OR “Park Exercise” OR “Park Training”) AND (Adult OR “Young Adult” OR “Healthy Adult” OR “Older Adult”) AND (“Health Outcomes” OR “Anthropometric Outcomes” OR “Anthropometric characteristics” OR “Anthropometrical outcomes” OR “Anthropometrical characteristics” OR “Wellbeing” OR “psycho-social Wellbeing” OR “Quality of Life” OR “Physical Performance” OR “Physical Fitness”).

Moreover, hand searches of key conference proceedings, journals, and professional organizations’ websites were conducted by SM, AG, and PML, and, in accordance with the snowball technique, references cited in the primary papers were examined to discover possible additional papers.

### 2.2. Quality Assessment and Data Extraction

Screening and checking phases followed different steps. First of all, the reviewers (SM, AG, MM, and PML) independently and blindly screened eligible papers after the removal of duplicates, reading titles, and abstracts to select pertinent papers. After the first screening, the reviewers (SM, AG, and PML) retrieved and read the full text of all potentially eligible studies. Disagreements about the eligibility of the studies for inclusion were resolved through discussion between all the researchers’ groups, and if more information was necessary, the study authors were contacted. Finally, the investigators, following the standardized rules for literature collection given by the Cochrane Reviewers handbook [28], independently obtained the information of the included studies focusing on the following characteristics: author, country, study design, population, intervention, outcomes, and results.

The studies included in the final step were independently and separately evaluated for the risk of bias by researchers (AG and SM) using the “A revised Cochrane risk of bias tool for randomized trials” (RoB 2) [29] and “The Risk Of Bias In Non-randomized Studies—of Interventions (ROBINS-I) assessment tool” [30]. Any disagreement between the quality scores separately assigned by the blind reviewers was resolved through discussion, and, if necessary, two more blind reviewers belonging to the research team (MM and ST) were involved as tiebreakers. This methodological choice was supported by the PRISMA guidelines [27].

RoB-2 tool analyzes different biases in five domains: (1) bias resulting in the randomization process; (2) bias arising from deviations from intended interventions; (3) bias linked to missing outcome data; (4) bias in the measurement of the outcome; and (5) bias on the reported result. The response options for the reported questions in each domain are as follows: yes, probably yes (PY), probably no (PN), no, and no information (NI).

These categories provide the possibility to assess an overall risk-of-bias judgment for the specific study result being evaluated in low risk of bias, some concerns, and high risk of bias.

The ROBINS-I scale uses seven different domains: (1) bias arising from confounding; (2) bias in the selection of the study’s participants; (3) bias in intervention classification; (4) bias linked to deviations from intended interventions; (5) bias resulting to missing data; (6) bias due to the measurement of outcomes; and (7) bias on the reported result. The response options for the domain level were the same as those of RoB-2, but the overall risk-of-bias judgment includes low risk, moderate risk, serious risk, and critical risk of bias.

## 3. Results

### 3.1. Study Selection and Characteristics

**Figure 1:**
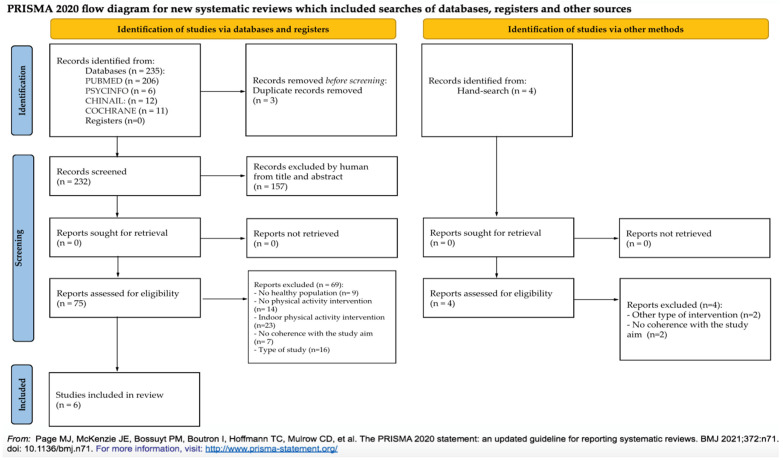
PRISMA 2020 flow diagram of studies selection [31].

Through database browsing and hand-searching, a total of 239 articles were identified (Figure 1). Considering the articles identified from databases, three were excluded because they were duplicated, and 157 were excluded after the reading of the abstract. Then, the authors read the full text of the articles, and 69 were excluded because they matched the exclusion criteria; finally, only six were considered relevant. All the records identified from hand-searching were excluded after reading the full text.

The main reasons for exclusion in the first step (abstract reading) were as follows: no physical activity intervention was carried out in the study, and no healthy people were involved. After the full-text reading (considering both reports from databases and hand-searching), the main causes of exclusion were the implementation of physical activity intervention carried out indoors and types of the study (without original primary data).

### 3.2. Risk of Bias Assessment

Each study was evaluated for quality assessment differentiating RCTs from quasi-experimental studies. The five studies categorized as RCTs scored a risk of bias from low to some concern, as shown in the Table 2 showing studies that resulted in low risk [32,33,34,35,36] of bias and two with some concerns [34,35,36,37].

Considering the quasi-experimental study performed by Song et al. [32,33,34,35,36,37], the response to the quality assessment was moderate concerns.

The major concerns were related to the second domain (risk of bias due to deviations from the intended interventions) mainly because there were no blinding of participants and people delivering the intervention given that it concerns physical activity practice (item #2.2–2.3).

### 3.3. Data Extraction of the Included Study

Table 3 summarizes the principal aspects and results of the included studies evaluating the effects of PA interventions on health outcomes in healthy people over 18. The geographic origin of the studies was as follows: Australia (*n* = 2), Korea (*n* = 1), Japan (*n* = 1), Spain (*n* = 1), and Singapore (*n* = 1). Study characteristics were heterogeneous. The sample size varied from 23 to 160 people. Ages ranged from 22 to 80 years.

We extracted the intervention characteristic by adopting the “F.I.T.T.” classification (frequency, intensity, time, type) mainly used in exercise prescription [38].

The duration of the experimental design varied from 3 days to 20 weeks and the frequency from two to seven times a week. The “type” of the intervention included two studies involving walking intervention [36,37], two studies involving a combination of resistance training and aerobic were used [33,35], and two studies involved resistance training [32,34]. According to this, the outcomes were heterogeneous, varying from performance tests such as balance test and handgrip test to well-being and quality of life assessed through a questionnaire. Table 3 describes the details of the included studies.

## 4. Discussion

The present systematic review was aimed at investigating the existing evidence regarding the effects of PA interventions carried out in GBS involving healthy people (aged ≥ 18 years). While research has previously assessed how GBS affects health and physical well-being, the relationship between exposure and health effects for healthy people is not well-known. For this reason, the aim of the present systematic review was to investigate the existing evidence regarding the effects of PA interventions performed in a natural environment involving healthy people (aged ≥ 18 years).

The systematic research of the literature found six studies, with different kinds of interventions and outcomes. The six eligible studies were scored as of medium quality and showed several improvements in health outcomes, which will be investigated in the present section.

Among the PA interventions adopted in the included studies, walking is a cost-effective one, which might appeal to most of the population [39]. In connection with this, walking in a blue space and in an urban park showed better well-being and mood responses compared with walking in an urban space or resting in a control site [32,36,37]. Song et al. (2015) reported significant differences in the questionnaires administered during the study [32]. In fact, the SD score was higher after walking in an urban park for three adjectives: comfortable, natural, and relaxed (*p* ≤ 0.05), and lower for the negative subscales tension–anxiety, anger–hostility, fatigue, and confusion (*p* ≤ 0.05). Finally, the positive mood state vigor was significantly higher for walking in the urban park than for the city-center walk (*p* ≤ 0.05). Song et al. (2015) did not report the effect size, but they used the Wilcoxon signed-rank test to analyze differences in psychological indices reported after walking in the two environments. Vert et al. (2020) found similar results for the blue space. In fact, participants showed better well-being and mood response after walking in a blue space versus an urban space or a control site (*p* ≤ 0.05). The WHO-5 total well-being score increased when participants were exposed to a blue environment (*p* ≤ 0.05). In addition, Vert et al. (2020) did not report the effect size, and they used mixed-effects regression models to evaluate the difference linked to the environment. One of the easiest ways to be and remain active is walking, and it is also the most popular [40]. A study found that adults who achieved the right amount of PA observed that walking was the most reported activity [40]. This could be due to its accessibility. Walking is a universal form of PA that a large part of the population can practice without differences in age, sex, education, income level, or ethnic group. Expensive equipment, special skills, or special facilities are not required in walking. There is an inverse association between the risk of developing coronary heart disease and overall walking in women [41]. In addition, walking is an important activity for older people. In fact, walking outdoors at least once a week has been associated with achieving more time spent in moderate to vigorous PA than walking indoors [42], and it also provides a way to take part in relevant activities, such as shopping or leisure activities (e.g., visiting friends or pleasure walking). Being physically active is linked to substantially lower costs of medical care [43], and, in particular for older adults where the risk of chronic disease is higher, walking has the potential to reduce medical expenditure [44]. In this review, two studies used walking as an intervention; they had similar objectives even if they used different questionnaires to evaluate the psychological answers to walking in a different kind of environment [32,36,37]. In particular, the first article aimed to value psychological and cardiovascular responses of the exposure to blue space, urban space, and a control site and to value whether well-being and mood effects were constant for (at least) four hours after exposure [36,37]. The second article would clarify the physiological effects of walking in urban parks during fall (autumn) [32,37]. Both articles found positive effects after the period of interventions, so they seem to suggest that walking in a natural environment had multiple positive effects. However, more studies are needed to improve the knowledge about this topic.

Concerning performance outcomes, resistance training induced significant improvement in body muscular strength, aerobic fitness, number of steps, functional mobility, systolic blood pressure, and waist circumference in the experimental group than in the control group [33,34,35]. In two studies, outdoor fitness equipment was used [32,33,35]. Several studies showed that outdoor fitness equipment has become very popular worldwide in numerous green and blue spaces and built-up environments [45,46,47,48,49]. Outdoor fitness equipment (OFE) can be used by a large part of the population (there is also OFE adapted for people in wheelchairs) because it provides free access to fitness training for the community and also enables different kinds of training (e.g., resistance training or circuit training) [42,48,49]. The results of the use of outdoor fitness equipment are mixed. Sales et al. found significant improvements in knee strength, balance, 2 min walk test, and sit to stand [31]. Meanwhile, Kim et al.’s study showed significant improvement in the upper-body strength. These differences in the results are linked to the different kinds of outdoor fitness equipment used. In fact, there are different manufacturers that design and produce outdoor fitness equipment, with differences in shape, materials, size, or smoothness of operation [6].

Plotnikoff et al. and Muller-Riemenshneider et al. evaluated the effects of PA in green and blue spaces and the effectiveness of face-to-face counseling [34,35,36]. In both studies, the participants received information about the parks in their city or their neighborhood to promote the use of a kind of environment. Plotnikoff et al. used a smartphone application, called eCoFit, in which the participants of the experimental group could find workout circuits suited for several geographical locations in the city. Indeed, in the study of Muller-Riemenshneider et al., the participants received an information brochure and a sheet where they filled in the types of activities they aimed to do each week over the trial period [34,36]. Even if the study design is similar between the two studies, the results are not comparable because they used different types of exercises and different questionnaires, and the time of the study was different. However, both studies found positive effects of the interventions. The experimental group in Muller-Riemenshneider et al.’s study was asked to join one hour of an outdoor structured and supervised PA program every week in a park. The control group received only standard PA promotion materials. At the end of six months, the experimental group had a significant increase in recreational PA, time spent in parks, and PA in parks. Additionally, they achieved improvements in chosen measures of quality of life and well-being, especially the psychological quality of life. Plontikoff et al.’s study divided into two parts the design for the intervention group: for 10 weeks, the participants performed personal sessions and used the app for smartphones; then, for the other 10 weeks, they used only the smartphone app. Most of the improvement related to health outcomes at 10 weeks was also confirmed at 20 weeks. This suggests that the participants continued the PA during phase 2. One of the key objectives was to promote the use of local green spaces, and eliminating many of the common barriers to participating can be interesting to verify if, after a longer period (a year), the participants of the intervention group continued to use the green spaces as a place to do PA. Despite several pieces of evidence on the health benefits of green and blue spaces, they are generally underused [21,50], so it can be important to sensitize the population more about the potential of this kind of environment.

From a public health perspective, these results can represent a strategy to be implemented to make the most of the natural setting to amplify the benefits of physical activity practice with a view to preventing health risks and saving resources. In connection with this, a recent systematic review recommends for future policies and research to take a more integrated multisystem approach and be inclusive of local and spatial authority planning and meet the needs of transport and natural resources [9].

The concept of “blue space” has not been widely used compared with green space, even if some studies demonstrated the potentially higher effects of blue space on people’s health. For this reason, it would be important that future studies propose physical activity programs in blue spaces to more consistently verify the benefits of this type of environment.

## 5. Conclusions

In conclusion, the current systematic review found that physical activity interventions including exercise and simple activities, carried out in an outdoor green–blue space natural environment, can have a positive impact on a healthy population, both after a few weeks of intervention or after several weeks, and can be an effective strategy to enhance and promote healthy lifestyles.

No exhaustive conclusion can be drawn based on available evidence. However, this systematic review highlighted the need to extend this kind of intervention that might stimulate a change in adults’ lifestyles, involving also their mood and job spheres. This approach can represent future effective integrative strategies to gain more benefits by practicing physical activity in natural green and blue spaces.

## Figures and Tables

**Table 1:** PICOTS criteria for eligibility.

Parameter	Inclusion Criteria	Exclusion Criteria
Population	Healthy people Age range: adult (aged ≥ 18 years)	People with acute or chronic conditions People aged under 18
Intervention	Outdoor PA intervention is carried out in the natural environment and natural and mixed settings (specifically green and blue spaces)	Absence of PA intervention; indoor PA intervention
Comparator	Standard treatment No PA intervention Another type of PA intervention	
Outcome	Physical fitness Quality of life Intervention satisfaction evaluation The physical performance or other indices of physical performance Anthropometric characteristics and anthropometric evaluation	No information about PA effects
Timing	10-year publication date limit English language Full text available	Published before 2011 Not in the English language No full text is available
Study design	Experimental or observational study with original primary data	Study protocol or other studies without original data

Note: PA—physical activity.

**Table 2:** Quality assessment of RCTs and quasi-experimental studies.

Authors	Study Design	Tool for Assessment	Quality
Song et al., 2015 [32]	Quasi-experimental	ROBINS-I Scale	Moderate concerns
Sales et al., 2017 [33]	RCT	RoB2 Tool	Low risk
Plotnikoff et al., 2017 [34]	RCT	RoB2 Tool	Some concerns
Kim et al., 2018 [35]	RCT	RoB2 Tool	Low risk
Muller-Riemenschneider et al., 2020 [36]	RCT	RoB2 Tool	Low risk
Vert et al., 2020 [37]	RCT	RoB2 Tool	Some concerns

RCT: randomized control trial; ROB2: Cochrane risk-of-bias tool for randomized trials; ROBINS-I: the Risk Of Bias In Non-randomized Studies—of Interventions.

**Table 3:** Characteristics of studies included.

Study	Study Design	Population	Intervention	Outcome	Results
Song et al. 2015, Japan [32]	NRCT	N: 23 men (aged 22.3 ± 1.2, height 171.1 ± 4.7 cm, weight 63.4 ± 8.1 kg, BMI 21.5 ± 2.1 kg/m^2^)	Type: 15 min of walking in two different environments—an urban park and a city area; after walking, the subject returned to the waiting room and completed the questionnaires. Participants rested for approximately 20 min and repeated the experiment in the other environment. There were no significant differences in the average speed between the two environments. Frequency: twice a day Time: 3 days	Physiological relaxation, three different questionnaires were used to investigate the psychological responses after walking in each site. The questionnaires were the SD scores, POMS, and STAI score. Heart rate and its variability were measured to investigate automatic nerve activity i.	The participants showed statistically significant differences in their physiological and psychological responses to the walking in different environments. The natural logarithm of the HF component, which is an estimate of the parasympathetic nerve activity, was higher when subjects walked in the urban park than when they walked in the city area. The mean ln(HF) was significantly higher in the urban-park walking than city-area walking (*p* ≤ 0.01). Then, the estimation of the sympathetic nerve activity was lower during the urban-park walking than city-area walking. The mean heart rate was significantly lower in the urban-park walking than city-area walking (*p* ≤ 0.01). A significantly higher SD score was observed following the urban-park walking than those following the city-area walking for the three adjectives: comfortable, natural, and relaxed. The negative subscale of tension–anxiety, anger–hostility, fatigue, and confusion was significantly lower after walking in the urban park than walking in the city area (*p* ≤ 0.05). On the contrary, the positive mood state vigor was significantly higher for walking in the urban park (*p* ≤ 0.001). The total STAI score was 19.3% significantly lower after walking in the urban park than after walking in the city area (*p* ≤ 0.01)
Sales et al. 2017, Australia [33]	RCT	N: 48 CG: 21 (age 70.2 ± 8.2, 77% women, BMI 28.1 ± 5.0, 6% current smoker, 29% ex-smoker, 52% daily alcohol assumption, 61.9% had previous falls history, 47.6% had falls over 12 months) EG: 27 (age 75.1 ± 7.9, 64% women, BMI 28.9 ± 5.3, 3% current smoker, 42% ex-smoker, 41% daily alcohol assumption, 62.9% had previous falls history, 40.7% had falls over 12 months)	Type: different kinds of outdoor exercises with different exercise stations: push-ups, modified pull-ups, balance stool, sit to stand, ramp + net + climb through, balance beam, steps, step-ups or taps on platform, gangway, calf raises + finger steps, round snake pipe, sharp snake pipe, hip extension, screws and turners, and hip abduction. Exercisers were paired in stations, and an exercise session could include up to eight stations. Frequency: two times a week, approximately 1–15 h, with 5–10 min of warm-up, followed by 45–75 min on the equipment station and 5–10 min of cool-down exercises. Time: 18 weeks of interventions	BOOMER test, to assess the effectiveness of the exercise park to improve balance; handgrip strength, to measure the physical strength; single leg test standing, to measure the static balance; 2 min walk test, to assess physical tolerance, functional mobility; 30 min sit-to-stand test, to evaluate the strength of the knee extender muscle; feasibility, defined as the number of participants recruited and retained over the recruitment period; physical composite scores, shortfalls efficacy scale international, numbers of falls over 12 months	No significant improvement in the BOOMER test (CG, 13.5 ± 1.7 pre, 13.9 ± 1.4 post, *p* = 0.6 EG 13.6 ± 1.4 pre, 13.7 ± 1.3 post, *p* = 0.6, *p* between groups = 0.4) and the improvement in quality of life (CG 49.1 ± 7.91 pre, 48.9 ± 7.6 post, *p* = 0.2, for the physical component, 51.4 ± 6.1 pre, 51.6 ± 7.9 post, *p* = 0.6, for the mental component; EG 46.9 ± 7.6 pre, 49.6 ± 8.3 post, *p* = 0.4, for the physical component, 53.1 ± 9.8 pre, 54.5 ± 7.0 post, *p* = 0.6, for the mental component) and falls efficacy (CG 11.3 ± 4.0 pre, 10.9 ± 3.7 post, *p* = 0.4, EG 10.3 ± 3.4 pre, 9.3 ± 2.5, post, *p* = 0.4, *p* between groups = 0.1). EG showed significant improvements in knee strength (84.2 ± 36.5 pre, 96.4 ± 44.4 post, *p* = 0.01), balance (single leg stance, 15.6 ± 11.0 pre, 17.3 ± 11.3 post, *p* = 0.01), 2 min walk test (140.6 ± 30.5 pre, 152.1 ± 28.7 post, *p* = 0.01), and sit to stand (10.5 ± 3.0 pre, 12.1 ± 2.7 post, *p* = 0.01). Regarding feasibility, 87% of EG completed the 18-week intervention with mean attendance to the session of 79.6% and 14% of the CG attended the social meeting offered.
Plotnikoff et al. 2017, Australia [34]	RCT	N:84 (aged 44.7 ± 14.0, BMI 33.3 ± 5.7 kg/m^2^) CG: 42, aged 45.1 ± 14.7, BMI 31.7 ± 5.1 kg/m^2^, EG: 42, aged 44.2 ± 13.5, BMI 35.0 ± 5.9 kg/m^2^	Type: EG—five face-to-face group intervention, each intervention lasted for 90 min and consisted of 30 min of cognitive group and 60 min of small group outdoor training and outdoor PA with the eCoFit smartphone app that included workout circuits, and a description of where and how to use an outdoor physical environment to be more physically active. CG: no interventions Frequency: once a week Time: 20 weeks of interventions; phase 1: 1–10 weeks face-to-face group intervention; phase 2: 11–20 weeks eCoFit smartphone app	Aerobic fitness to assess aerobic fitness; lower body muscular fitness using the chair stand test; steps/day measured using pedometers; functional mobility using the Timed Up and Go test; waist circumferences, BMI, and systolic and diastolic blood pressure	After 10 weeks, EG improved aerobic fitness (4.50 mL/kg/min), the strength of the lower body, numbers of steps (1330 steps), mobility (−1.8 s), and systolic blood pressure, and there was a decrease in waist circumference (−2.8 cm). After 20 weeks, EG showed effects on the upper and lower body strength, blood pressure, and functional mobility. Survey conducted at the end of the intervention showed positive feedback for group cognitive session, outdoor training, and use of the eCoFit app.
Kim et al. 2018, South Korea [35]	RCT	N: 35 (aged 73.20 ± 4.90, women characteristics (32): BMI 25.48 ± 2.41, kg/m^2^ height 151.98 ± 5.90 cm, weight 58.73 ± 8.19 kg, lean mass 19.64 ± 2.50 kg, body fat 36.84 ± 3.36%; men characteristics (3): BMI 24.70 ± 2.87 kg/m^2^, weight 69.40 ± 8.39 kg, 168.20 ± 4.75 cm, lean mass 27.00 ± 3.72 kg, body fat 28.66 ± 3.95%) RC: 12, CoG: 13 CG: 10	Type: RC—outdoor resistance training using leg extension, pull weight, chair pull, for a total of 50 min of training; CoG: outdoor aerobic and resistance training using leg extension, pull weight, chair pull, sky-walker, cross-country, for a total of 70 min; CG: no interventions Time: 6 weeks of interventions at different intensity evaluated with the Borg scale	Fitness was evaluated with five fitness tests designed for the elderly (30 s chair stand, 30 s arm curl, 244 cm up and go, one-leg stand, and 2 min step), as well as number of pushups and 6 min walking	Improvement in upper-body strength in both groups (RC 19.16 ± 11.40 pre, 30.16 ± 13.13 post; CoG 11.07 ± 9.62 pre, 22.23 ± 12.95 post); lower-body endurance was higher in the CoG (561.84 ± 67.22 m) than the CG (486.44 ± 96.14 m).
MullerRiemnschneider et al. 2020, Singapore [36]	RCT	N: 160 (aged 51.1 ± 6.3, 127 women, total MVPA 442.7 ± 534.7 min/week) EG: 80 (aged 52.1 ± 6.5, 65 women) CG: 80 (aged 50.0 ± 6.0, 62 women)	Type: EG—face-to-face counseling on PA; they completed a park prescription sheet where they committed to a goal that specified the frequency, intensity, time, and location of exercise parks. Participants received two brochures developed for the trial: one provided information on the main parks and their different features, including walking trails and location of fitness corners. The second was generally about the Singapore National Park s Board. + invitation to weekly exercise sessions in parks; in addition, participants received half-way through the trial a brief counseling phone call to assess progress and included modification of the goal if necessary. CG: continued their daily routine; they received standard PA materials. Time: 6-week intervention.	Time spent on MVPA measured by an accelerometer and by questionnaire, total volume of PA, time spent on light and sedentary activity, time spent at the park, physical activity at the park, recreational MVPA, mental well-being (measured by SF-12, K-10, WHO5, and WHOQOL-BREF).	No differences between EG and CG were observed with regard to physiological distress and overall quality of life. The only difference was found for the psychological quality of life, which was higher in EG than in CG (*p* = 0.047). The difference was not statistically significant regarding the mean differences in MVPA among participants. EG showed a significant increase in the time of recreational PA (EG 142 ± 155.4 min/week, CG 93.6 ± 131.0 min/week, *p* = 0.044), time spent in parks (EG 333.9 ± 506.2 min/month, CG 186.4 ± 85.4 min/month, *p* = 0.047), and PA in parks (EG 333.0 ± 499.3 min/month, CG 140.5 ± 270.7 min/month, *p* = 0.005).
Vert et al. 2020, Spain [37]	RCT	N: 49 (aged 29, min 19, max 49, 69.5% women, BMI 22.6 ± 3.5 kg/m^2^, 88.1% saw blue space at work, 89.9% met the PA of WHO guidelines)	Type: for each study week, each participant was assigned to a different environment (blue, urban, or control site). All participants were exposed to all environments upon completion of the study. They walked 20 min in blue, urban, or control site. Participants were distributed in two turns: the first started at 10.00 a.m. and the second at 11.30 a.m. Frequency: 4 days a week Time: 3 weeks intervention	Participants completed a set of questionnaires (SWB, WHO-5, TMD, 4SDQ, and SF-36) to assesses their well-being, mood, and psychological responses, before and after each walking. In addition, sleep characteristics and general health were assessed. Blood pressure, pulse rate, and heart rate variabilities were continuously measured before and after the walking.	Better well-being and mood responses after walking in a blue space versus an urban space or control site (*p* ≤ 0.05). For SWB, no significant differences were found. For WHO-5, the “total well-being score” was increased when participants were exposed to blue environment (*p* ≤ 0.05). TMD was significantly lower for the negative subscales after walking along the blue route compared with urban space and control site (*p* ≤ 0.05). 4SDQ did not show significant differences between the environments. Statistically significant increase was found in systolic blood pressure and pulse rate in the blue and urban environments compared with the control site. Increase in SNS activity during and after walking in blue and urban spaces.

RCT: randomized control trial; NRCT: nonrandomized control trial; N: numbers of participants; CG: control group; EG: experimental group; RC: resistance group; CoG: combined group; PA: physical activity; BOOMER: Balance Outcome Measure for Elder Rehabilitation; min: minutes; SD: semantic differential; POMS: profile of mood state; STAI: state–trait anxiety inventory, MVPA: moderato-to-vigorous physical activity; PA: physical activity; SWB: subjective well-being; TMD: total mood disturbance; 4SDQ: four-dimensional symptom questionnaire.

## Data Availability

The data presented in the study are included in the article.
